# Development and evaluation of a PCR assay for rapid detection of azithromycin resistant *Campylobacter* isolated from diarrhoeal patients in Kolkata, India

**DOI:** 10.1186/s13099-017-0186-9

**Published:** 2017-06-21

**Authors:** Piyali Mukherjee, Shanta Dutta, Asish K. Mukhopadhyay

**Affiliations:** Division of Bacteriology, National Institute of Cholera and Enteric Diseases, P 33, CIT Road, Scheme XM, Beliaghata, Kolkata, 700010 India

**Keywords:** *Campylobacter*, Azithromycin resistance, Campylobacteriosis, PCR assay

## Abstract

**Background:**

*Campylobacter* is a well-known bacterial pathogen for triggering acute gastroenteritis in humans both in developed and developing countries. This organism is highly resistant to fluoroquinolones. Macrolides are very much useful for the treatment of campylobacteriosis when clinical therapy is necessary. However, increasing resistance to azithromycin, a potent macrolide has been reported in *Campylobacter* in recent years. Macrolide resistance in *Campylobacter* is found mainly due to point mutation in V region of 23S rRNA.

**Results:**

We have developed a PCR based assay, which can detect the azithromycin resistant and sensitive *Campylobacter* strains utilizing mutation responsible for the phenotype. This PCR was validated using 359 *Campylobacter* strains isolated from diarrhoeal patients at Kolkata, India. Antimicrobial resistance through disk diffusion method was also performed on these strains as a gold standard. Studies through sequencing analysis further confirmed the PCR result.

**Conclusion:**

This study describes a simple and rapid method for detection of mutation conferring macrolide resistance with additional feature of identification of sensitive strains.

## Background

*Campylobacter* species, particularly *Campylobacter jejuni*, have been documented as an important pathogen for causing acute bacterial gastroenteritis in humans. It is projected that around 400–500 million cases of diarrhea are caused by the *Campylobacter* sp. each year, worldwide [1]. In food borne diarrheal illness, *Campylobacter* stood second etiologic agent among the enteric pathogens [2]. *Campylobacter* are part of normal enteric flora of a wide range of domestic animals and poultry as well as wild animals and birds [3, 4]. So, *Campylobacter* can be transmitted to humans mainly through the consumption of contaminated foods of animal origin, especially undercooked poultry meat, unpasteurized milk and dairy products, as well as by ingestion of other foods that are cross-contaminated by raw poultry meat during food preparation. Though *Campylobacter* mediated gastroenteritis is self-limiting; antibiotic therapy is needed to reduce severity of disease. The most common drugs used to control *Campylobacter* mediated infections are fluoroquinolones and macrolides. High resistance towards fluoroquinolones has shifted the treatment towards macrolides [5]. Generally, the prevalence of macrolide resistance among *Campylobacter* strains (including both *C. jejuni* and *Campylobacter coli*) isolated from humans, broilers and cattle in the USA and Canada has been reported at around 10% [6]. In contrast, more than 40% of *C. coli*, isolated from turkeys and swine in the USA, were resistant to this antimicrobial agent [6]. Likewise, macrolide resistance among *Campylobacter* isolates from humans and *C. jejuni* isolates from chickens and cattle has been low and stable in most European countries, especially in Scandinavia, but a high prevalence of macrolide resistance, ranging from 15 to 80%, was observed in *C. coli*, isolated from chickens and swine [6, 7]. A tendency for increased macrolide resistance in *Campylobacter* has been reported in different developing countries which include Asian countries also [8, 9]. In northern part of India, the macrolide resistance was 6.1% during 2005 [10] and reached 22.2% in 2013 [11] whereas, 0.7% macrolide resistance was reported in eastern India during 2008–2010 [5] and increased to 4% during 2010–2012 [12]. Studies from Pakistan stated that macrolide resistance of *Campylobacter* was increasing alarmingly—from almost 0% (2002) to 27% (2011–2012) in human isolates [13, 14]. *Campylobacter* isolates from Poultry in Pakistan were found more resistant towards macrolide [14]. *Campylobacter* isolates from human in Bangladesh were found highly susceptible to macrolides (0.5% during 2005–2008) [15]. The situation is similar in China. Erythromycin resistance is low in human isolates (1–2%) whereas high in chicken and swine isolates (18% and 37.9 to 54.7%) [9, 16, 17]. Reports from Sub-Saharan-Africa demonstrated low level of resistance towards macrolides from human *Campylobacter* isolates but high level of resistance from cattle isolates [18–20]. On the other hand, high level of macrolide resistance has been reported among human clinical *Campylobacter* isolates from developed parts of Africa [7, 21]. Development and spread of resistance to macrolides among *Campylobacter* will significantly limit options for clinical treatment.

Macrolides interrupt protein synthesis in bacterial ribosome by targeting the 50S ribosomal subunit and inhibit bacterial RNA-dependent protein synthesis. Macrolide resistance in *Campylobacter* is found due to modification of the ribosome target binding site and not by target methylation or enzymatic drug modification seen in other bacterial species. Base substitutions at positions 2074 and 2075 in the V region of 23S rRNA gene (rrnB operon) in *Campylobacter* are the most common cause conveying erythromycin resistance and cross resistance to other macrolides. The most common mutation found to be transition of adenine to guanine at nucleotide position 2075 (A2075G) [7, 17].

Azithromycin, a macrolide is now widely used for the treatment of gastroenteritis and upper respiratory tract infection in India. Our recent study has indicated the growing level of resistance in *Campylobacter* isolated in India towards this antibiotic [12]. This study also indicated that azithromycin resistant *Campylobacter* isolates harboured A2075G mutation in 23S rRNA gene and is mainly responsible for high level of azithromycin resistance in Kolkata. During severe infection, reducing the time for detection of the resistance phenotype can be helpful to improve the condition of patient whereas; traditional disk diffusion method consumes at least 48 h to detect azithromycin resistance. We have developed and evaluated a PCR based assay, which is able to discriminate not only *Campylobacter* strains resistant to azithromycin but also the sensitive strains to that antibiotic.

## Methods

### Bacterial strains

Three hundred and fifty nine *Campylobacter* strains were used in the study. These strains were isolated from paediatric diarrheal cases (children of >5 years) in Kolkata, India during 2010–2015. These strains were either *C. jejuni* or *C. coli*. For standardization of the PCR, azithromycin sensitive *C. jejuni* subsp. *jejuni* NCTC11168 (ATCC 700819) and azithromycin resistant *C. jejuni* isolated from Kolkata (BCH 00521) were used as controls. The strains were generally grown on BHI agar (Brain Heart Infusion agar, BD-Difco) supplemented with 5% horse serum (Invitrogen) at 37 °C in microaerophilic atmosphere overnight.

### Preparation of template DNA

The DNA was extracted according to the standard protocol [22]. In short, one loop full of fresh cultures of *Campylobacter* were suspended in 200 µl TE buffer and mixed with equal volume of phenol–chloroform–isoamyl alcohol (25:24:1) and centrifuged. Aqueous layer was then mixed with equal volume of chloroform–isoamyl alcohol (24:1) and centrifuged. Upper aqueous layer was taken as a pure DNA in a fresh tube, kept at aliquots in −20 °C and used as template during PCR assay.

### PCR assay and sequencing analysis

Three primers were designed to perform the PCR assay. 23s rRNA-Campy-1912F was common for both the azithromycin sensitive and resistant strains. Two reverse primers 23s rRNA-Campy-2075 R and 23s rRNA-Campy-2074 N-Rev were designed to detect resistant and sensitive alleles respectively (Table 1). Two different PCRs were set for resistance and sensitivity identification. Each PCR was carried out in 10 µl of reaction mixture containing; 1× PCR buffer (GeNetBio, Korea); approximately 5–30 ng of genomic DNA, 0.2 µM each of the forward and reverse primers; 0.2 mM deoxynucleotides, and 0.5 U of ExPrime Taq DNA polymerase (GeNetBio, Korea). The PCR parameter was standardized as follows—initial denaturation at 95 °C for 1 min 30 s, followed by 25 cycles of denaturation at 95 °C for 25 s, annealing at 60 °C for 10 s, and extension at 72 °C for 20 s, and a final extension at 72 °C for 5 min.

**Table 1:** Sequence of the oligonucleotides used in the study

Used for	Primer	Primer sequence (5′ to 3′)	Amplicon size (bp)	Reference
MAMA PCR	23s rRNA-Campy-1912F	AGTAAACGGCGGCCGTAAC		This study
	23s rRNA-Campy-2074 N-Rev	GTAAAGGTCCACGGGGTCATT	183	This study
	23s rRNA-Campy-2075 R	GTAAAGGTCCACGGGGTCAC	183	This study
Sequencing	F2-Campy-23S	AATTGATGGGGTTAGCATTAGC	552	21
	2420R-Campy-23S	AGAACCACCGGATCACTAAGA		10

Sequence analysis of V region of 23S rRNA of few representative strains was done by Sanger’s dideoxy method. For sequencing purpose, we used a 552-bp PCR amplified product of the V region of the 23S rRNA gene using primers mentioned in Table 1 [12, 23].

### Phenotypic assay

We followed the CLSI guideline for antimicrobial susceptibility test by disk diffusion method. In short, bacterial culture of 0.5 McFarland OD was spread onto Muller Hinton Agar plate containing 5% sheep blood and azithromycin disk (BD, Difco) was placed onto the plates. Plates were incubated at 37 °C in microaerophilic atmosphere for 48 h and reading was taken by measuring zone diameter. Determination of MIC was done for 12 azithromycin resistant strains using azithromycin E test strip (Biomerieux) by following manufacturer’s instructions.

### Statistical analysis

We followed conventional two-by-two table to calculate specificity, and sensitivity of the PCR assay. By convention, columns represent “Gold standard”, i.e. phenotypic identification of resistance results (resistant on left) and rows signify PCR assay results (resistant on top). If the PCR result showed positive, and was confirmed by gold standard, then it was marked true positive (TP) (indicated in left upper cell). In right upper cell, false positives (FP) were entered, i.e. resistant by PCR but phenotypically sensitive. In lower left cell, false negatives (FN) were entered—phenotypically sensitive, but PCR identified as resistant. In right lower cell true negatives (TN) were entered (both phenotypically and PCR sensitive). Sensitivity has been calculated by dividing true positive with sum of true positive and false negative. Specificity was calculated by dividing true negative with sum of true negative and false positive [24].

## Results

### Development of a mismatch amplification mutation assay PCR

In this study, we focused to develop a PCR based assay which can comprehensively discriminate azithromycin resistant and sensitive *Campylobacter* strains in a simple and rapid way. Two reverse primers 23s rRNA-Campy-2075 R and 23s rRNA-Campy-2074 N-Rev were designed to detect resistant and sensitive alleles respectively. The ‘23s rRNA-Campy-2075 R’ bears specific nucleotide C at 3′ end to detect 2075G mutation. An enhancing effect was added following introduction of change in nucleotide to mismatch at the second nucleotide position (i.e. the 2074th nucleotide) from the 3′ end of the primer. The other primer ‘23s rRNA-Campy-2074 N-Rev’ was carrying two mismatches in second and third nucleotide position from 3′ end with respect to resistant 2075G mutation and only single mismatch with respect to sensitive strains.

The designed primers were tested using DNA template from the control strains. Amplification using the resistant specific primers (23s rRNA-Campy-1912F and 23s rRNA-Campy-2075 R) yielded a 183-bp amplicon from the azithromycin resistant strain (BCH 00521) containing the A2075G mutation but no amplicon was found from sensitive strain NCTC 11168. On the other hand, PCR assay with the sensitive specific primers (23s rRNA-Campy-1912F and 23s rRNA-Campy-2074 N-Rev) produced 183-bp amplicon using template DNA from the sensitive strain NCTC 11168, while failed to provide any amplicon using template DNA from the resistant strain.

### Validation of the PCR assay

**Fig. 1:**
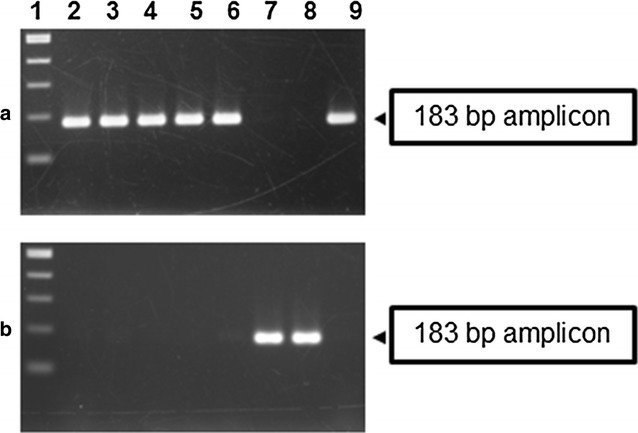
MAMA-PCR to detect the azithromycin resistant strains of Kolkata using primers 23s rRNA-Campy-1912F/23s rRNA-Campy-2075 R (**a**) and sensitive strains using primers 23s rRNA-Campy-1912F/23s-rRNA-Campy-2074 N-Rev (**b**) in representative *Campylobacter* strains of Kolkata. *Lane 1* contains a 100-bp size ladder. *Lanes 2* to *6* and *9*—representative resistant strains. *Lanes 7* and *8*—sensitive strains

Our newly designed PCR was evaluated using the template DNAs from 359 *Campylobacter* strains isolated between 2010 and 2015. Among the 359 *Campylobacter* tested, 24 strains yielded amplicons with the resistant specific primers but not with the sensitive specific primers (Fig. 1). On the other hand, remaining 335 strains produced amplicons with the sensitive specific primers but not with the resistant specific primers. In addition, all the strains were subjected to disk diffusion assay for azithromycin and the result perfectly matched with the PCR assay. The strains which gave amplicon with primer-set responsible for resistant phenotype were found resistant with disk diffusion method.

Accuracy of the test was measured by a conventional two-by-two (2 × 2) table. The information was obtained by comparing the PCR result with the gold standard and summarized in Table 2. By using the table, we have calculated the sensitivity, specificity, positive predictive value and negative predictive value. In this evaluation, our PCR showed 100% sensitivity and specificity. Positive predictive value and negative predictive value were also 100%.

**Table 2:** Comparative analysis of sensitivity and specificity of PCR assay and conventional antibiotic sensitivity test

	Conventional sensitivity test		Total
	Resistant	Sensitive	
PCR assay			
Resistant	24	0	24
Sensitive	0	335	335
Total	24	335	359

Sensitivity = 24/(0 + 24) × 100 = 100%

Specificity = 335/(0 + 335) × 100 = 100%

Positive predictive value = 24/(0 + 24) × 100 = 100%

Negative predictive value = 335/(0 + 335) × 100 = 100%

The E test assay with azithromycin of 12 representative resistant strains indicated that these strains were resistant to 256 µg/ml azithromycin. To further confirm the PCR result, sequencing of the V region of 23S rRNA of representative resistant and sensitive strains were done. Mutation of A to G in 2075 nucleotide position of 23S rRNA was found to be associated with high level of azithromycin resistance and sequencing results with our strains indicated the same. The sequencing results revealed all resistant strains had the A2075G mutation in the V region of 23S rDNA while the sensitive strain had no mutation in that site [12] (GenBank Accession Numbers KX579735 to KX579740).

## Discussion

The phenotypic tests followed by sequencing demonstrated a complete correlation between the newly designed PCR assay and azithromycin resistance. The overall macrolide resistance found in this study was 6.7%. In eastern India, the resistance towards macrolides was found to increase every fast, from 0.7% in 2008–2010 [5] to 4% during 2010–2012 [12] to 10% during 2014–2015 as indicated in our study. There are contradictions over the disk diffusion method to be used for antibiotic sensitivity of *Campylobacter* [25]. Our study indicated that disk diffusion method properly identified sensitivity pattern with respect to azithromycin and MIC results of resistant strains indicated that Kolkata strains showed higher level of resistance towards azithromycin.

Children below 5 years of age are mostly affected by *Campylobacter* infection in India. Macrolide resistance is emerging in India. All the macrolide resistant *Campylobacter* isolates found to be highly resistant towards azithromycin. As a result, resistant bacteria will be growing in presence of antibiotics while susceptible one stop multiplying or die. Thus, resistant mutants outnumber sensitive bacteria and can spread rapidly in the population. In this alarming situation, the assay described here might be proved to be a useful tool for identifying macrolide resistance. This assay can act as a validation tool for itself as the method described here can also identify macrolide sensitive strains. Previous method as described by Alonso et al. [26] exploited mutational sequence divergence at nucleotide position 2074 and 2075 of 23S rRNA to identify *Campylobacter* strains resistant to macrolides. However, we described a novel assay to discriminate macrolide resistant as well as sensitive strains by utilizing allelic difference at nucleotide position 2075. This newly designed PCR method is significant as it is able to screen macrolide sensitive as well as resistant strains and therefore may help researchers from different parts of the world to find emergence and dissemination of macrolide resistance in *Campylobacter* isolates. We also compared our PCR assay with the one described by Alonso et al. using different methods of template preparation. Crude DNA prepared by the method described in this study and also prepared by boiled lysate method (which produce considerable amount of impurities compared to the phenol: chloroform method) was used in the comparison. In both the cases, the assay described by Alonso et al. gave non-specific resistant band with some sensitive strains while our assay gave only specific one (data not shown).

The above described PCR method is suitable to identify resistance to azithromycin (macrolide) conferred by mutation at 2075 nucleotide position of 23S rRNA gene. There are different other mechanisms—(a) mutation in ribosomal protein L4 and L22, (b) efflux pump, (c) presence of horizontally transferrable *ermB* gene, described in literature which might be responsible for macrolide resistance [17, 27] in *Campylobacter*. From this part of India, we could only detect mutation in 23S rRNA gene [12] and thus, our PCR worked with 100% specificity and sensitivity. If azithromycin resistance occurs due to other than the mutation in 23S rRNA gene, this PCR assay will not be able to identify it.

## Conclusion

PCR based assays are widely accepted as well as popular because they consume considerably lesser time and more simple instrumental set up while compared to DNA sequencing, real time PCR and others which require highly equipped machinery as well as longer period to get results. There are several other publications identifying mutation in 23S rDNA [28–31]. Our newly developed PCR method describes about a low cost method with additional feature of identification of azithromycin (macrolide) sensitive strains with resistant ones. The mutation described as target in this study is associated with high MIC to macrolide in *Campylobacter*. This study describes a simple method for identification of mutation conferring macrolide resistance and allows us to become aware of increasing high level of resistance to azithromycin (macrolide). Thus, quick detection of azithromycin resistance might be helpful in the management of infectious disease and also for developing the strategy to prevent the misuse of already resistant drugs.
